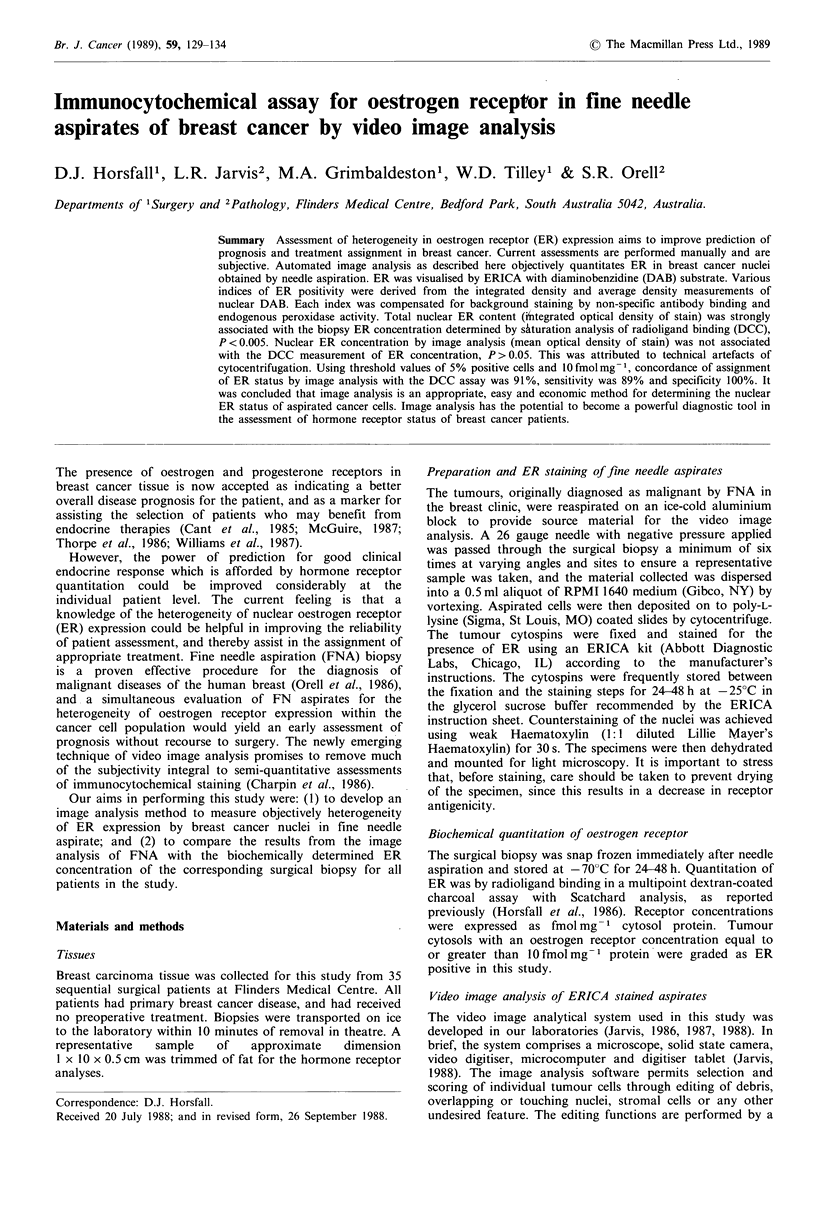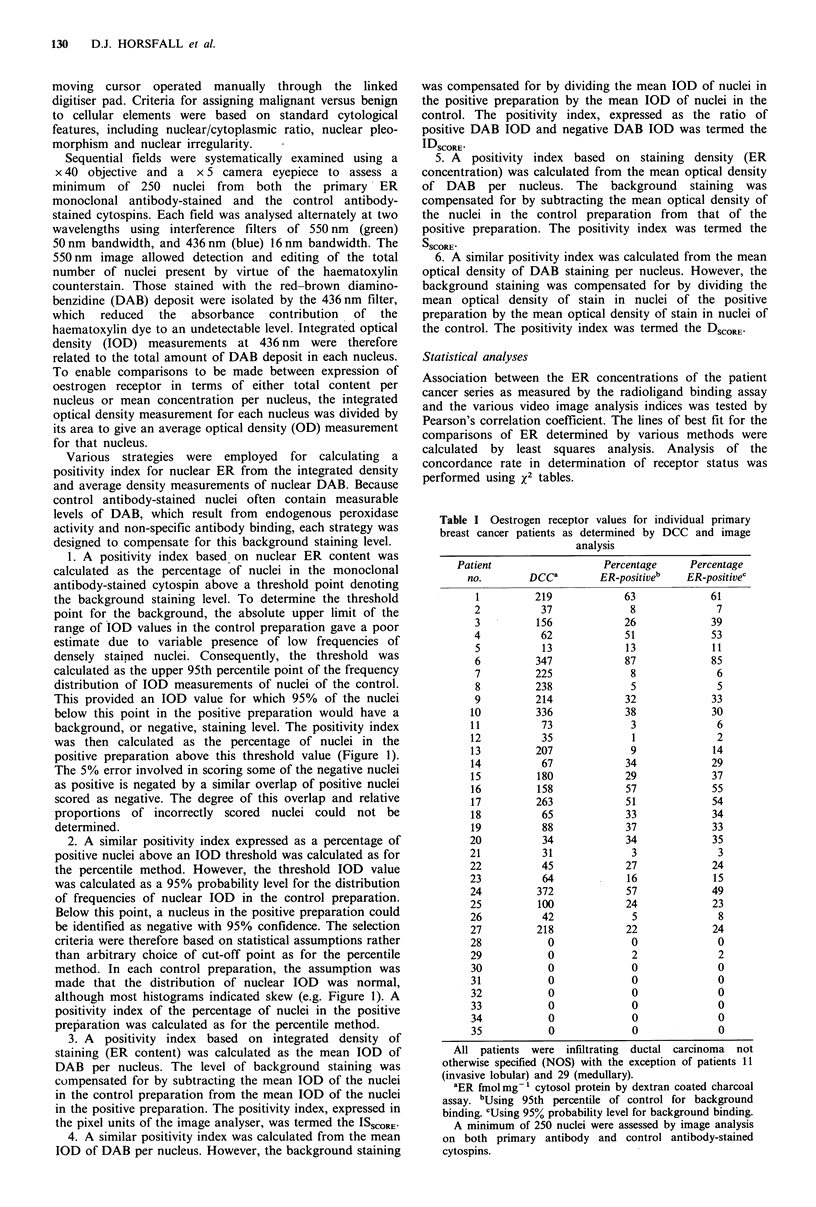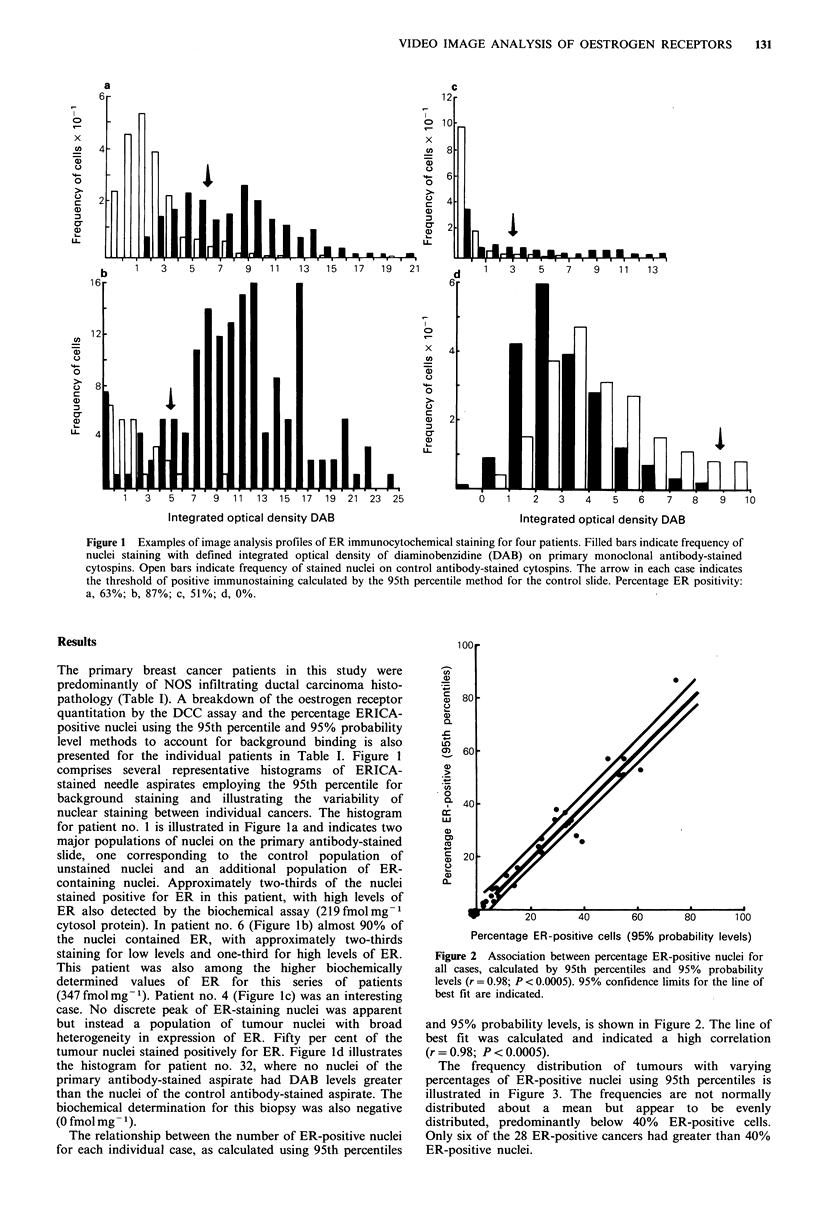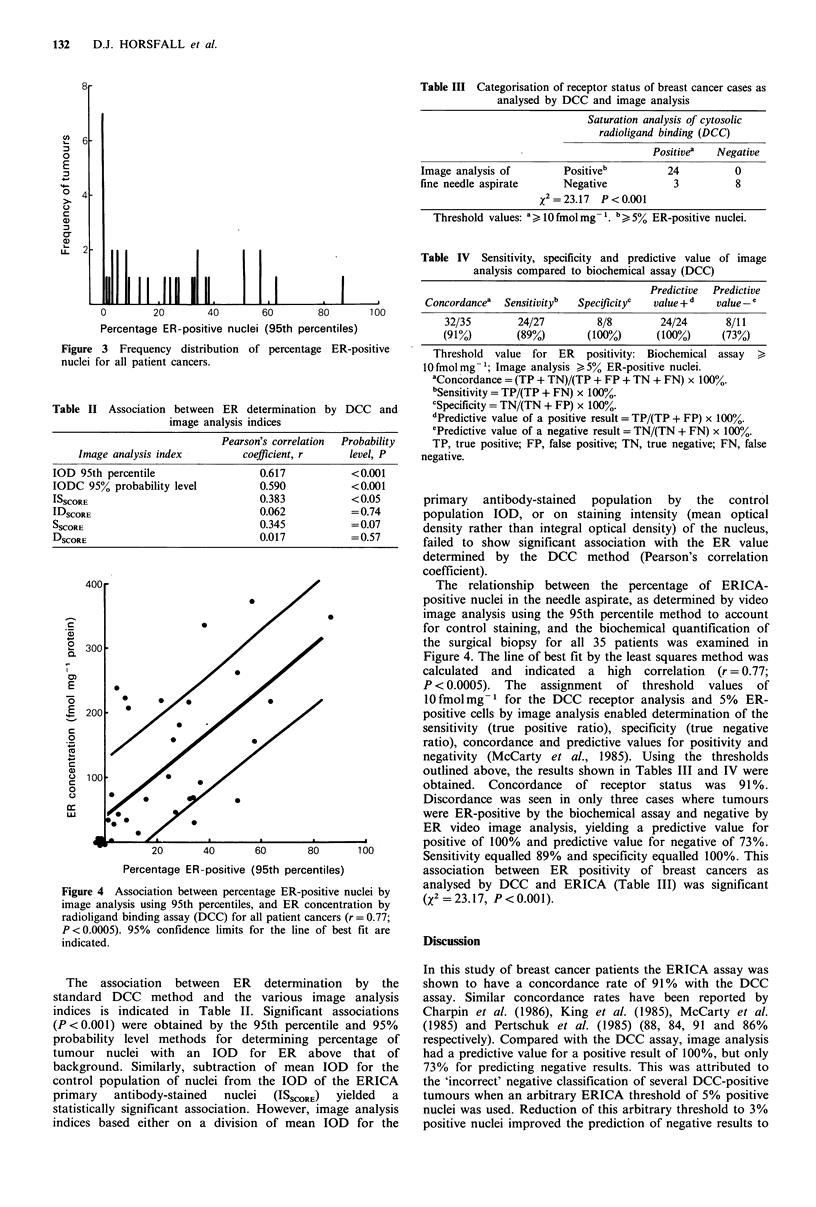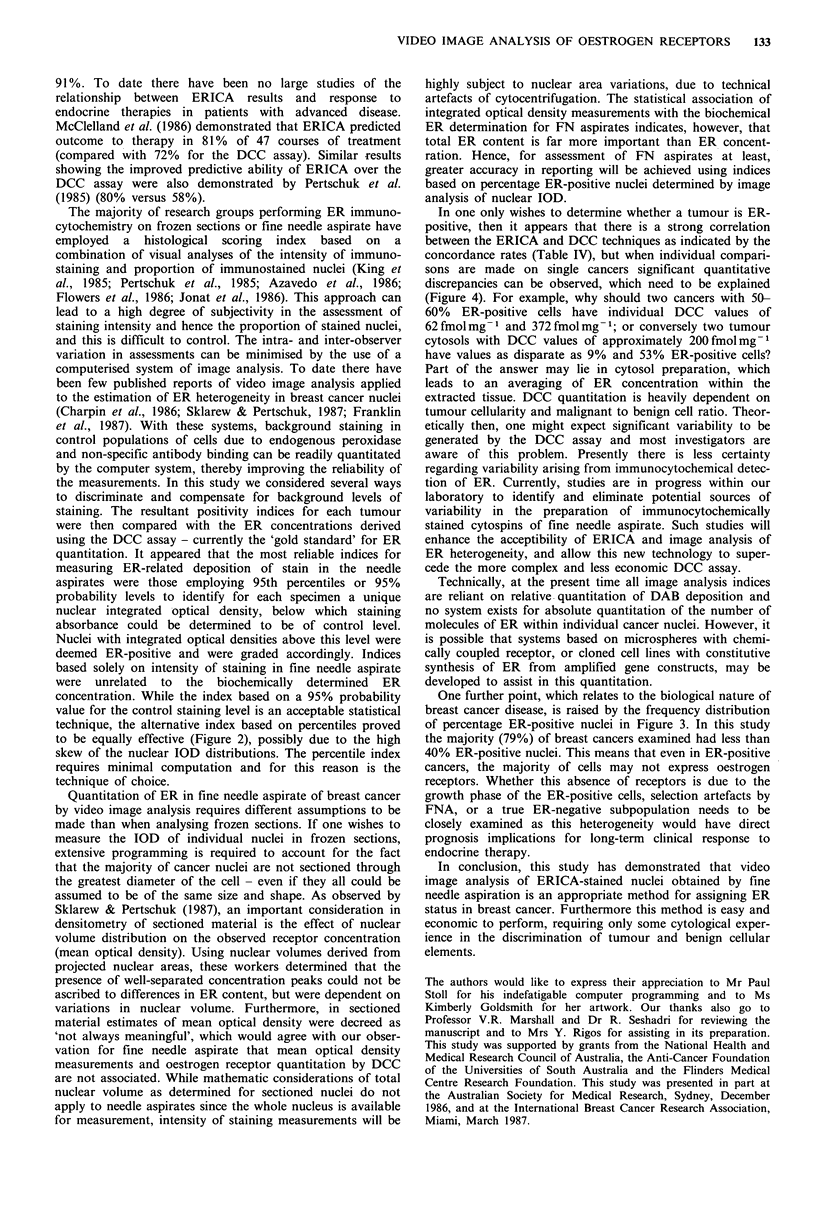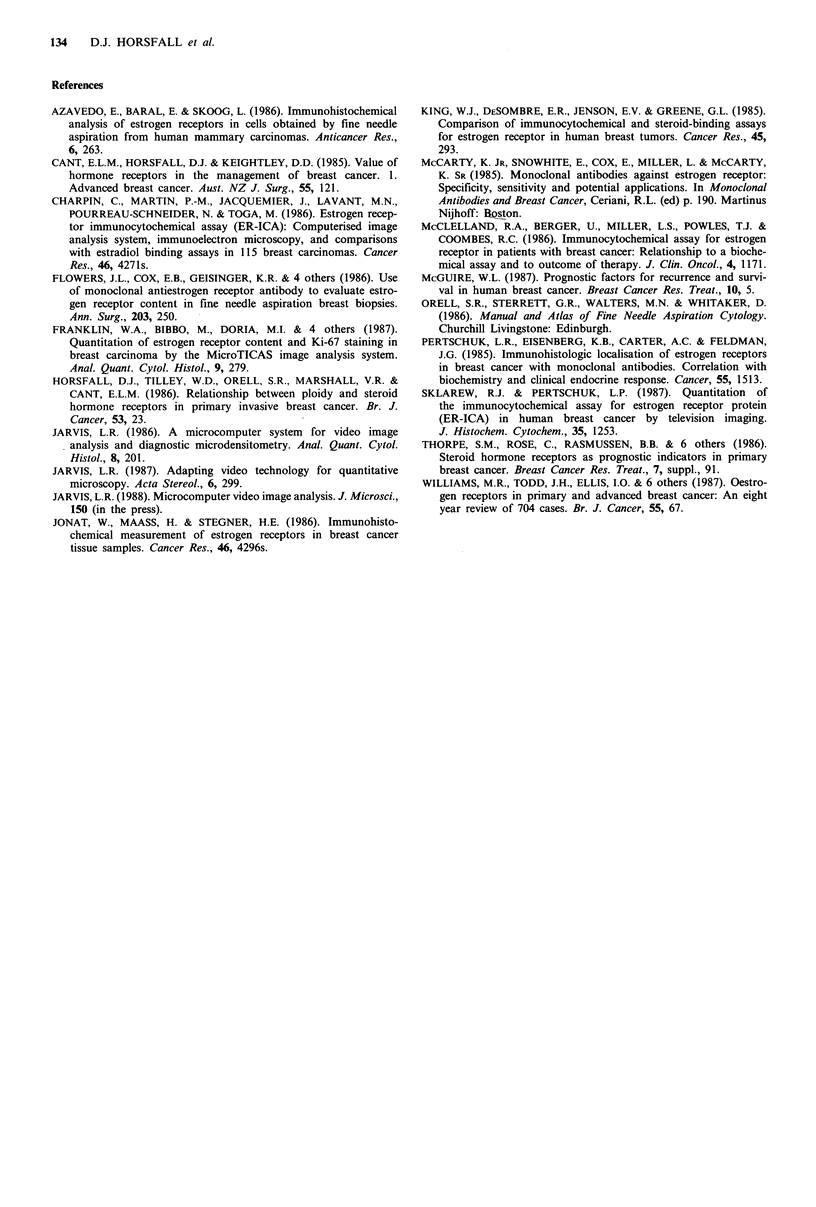# Immunocytochemical assay for oestrogen receptor in fine needle aspirates of breast cancer by video image analysis.

**DOI:** 10.1038/bjc.1989.26

**Published:** 1989-01

**Authors:** D. J. Horsfall, L. R. Jarvis, M. A. Grimbaldeston, W. D. Tilley, S. R. Orell

**Affiliations:** Department of Surgery, Flinders Medical Centre, Bedford Park, Australia.

## Abstract

Assessment of heterogeneity in oestrogen receptor (ER) expression aims to improve prediction of prognosis and treatment assignment in breast cancer. Current assessments are performed manually and are subjective. Automated image analysis as described here objectively quantitates ER in breast cancer nuclei obtained by needle aspiration. ER was visualised by ERICA with diaminobenzidine (DAB) substrate. Various indices of ER positivity were derived from the integrated density and average density measurements of nuclear DAB. Each index was compensated for background staining by non-specific antibody binding and endogenous peroxidase activity. Total nuclear ER content (integrated optical density of stain) was strongly associated with the biopsy ER concentration determined by saturation analysis of radioligand binding (DCC), P less than 0.005. Nuclear ER concentration by image analysis (mean optical density of stain) was not associated with the DCC measurement of ER concentration, P greater than 0.05. This was attributed to technical artefacts of cytocentrifugation. Using threshold values of 5% positive cells and 10 fmol mg-1 concordance of assignment of ER status by image analysis with the DCC assay was 91%, sensitivity was 89% and specificity 100%. It was concluded that image analysis is an appropriate, easy and economic method for determining the nuclear ER status of aspirated cancer cells. Image analysis has the potential to become a powerful diagnostic tool in the assessment of hormone receptor status of breast cancer patients.


Br. J. Cancer (1989), 59, 129–134 © The Macmillan Press Ltd., 1989

### Immunocytochemical assay for oestrogen receptor in fine needle aspirates of breast cancer by video image analysis

D.J. Horsfall[1], L.R. Jarvis[2], M.A. Grimbaldeston[1], W.D. Tilley[1] & S.R. Orell[2]

*Departments of [1]Surgery and [2]Pathology, Flinders Medical Centre, Bedford Park, South Australia 5042, Australia.*

**Summary** Assessment of heterogeneity in oestrogen receptor (ER) expression aims to improve prediction of prognosis and treatment assignment in breast cancer. Current assessments are performed manually and are subjective. Automated image analysis as described here objectively quantitates ER in breast cancer nuclei obtained by needle aspiration. ER was visualised by ERICA with diaminobenzidine (DAB) substrate. Various indices of ER positivity were derived from the integrated density and average density measurements of nuclear DAB. Each index was compensated for background staining by non-specific antibody binding and endogenous peroxidase activity. Total nuclear ER content (integrated optical density of stain) was strongly associated with the biopsy ER concentration determined by saturation analysis of radioligand binding (DCC), $P<0.005$. Nuclear ER concentration by image analysis (mean optical density of stain) was not associated with the DCC measurement of ER concentration, $P>0.05$. This was attributed to technical artefacts of cytocentrifugation. Using threshold values of 5% positive cells and 10 fmol mg$^{-1}$, concordance of assignment of ER status by image analysis with the DCC assay was 91%, sensitivity was 89% and specificity 100%. It was concluded that image analysis is an appropriate, easy and economic method for determining the nuclear ER status of aspirated cancer cells. Image analysis has the potential to become a powerful diagnostic tool in the assessment of hormone receptor status of breast cancer patients.

The presence of oestrogen and progesterone receptors in breast cancer tissue is now accepted as indicating a better overall disease prognosis for the patient, and as a marker for assisting the selection of patients who may benefit from endocrine therapies (Cant *et al.*, 1985; McGuire, 1987; Thorpe *et al.*, 1986; Williams *et al.*, 1987).

However, the power of prediction for good clinical endocrine response which is afforded by hormone receptor quantitation could be improved considerably at the individual patient level. The current feeling is that a knowledge of the heterogeneity of nuclear oestrogen receptor (ER) expression could be helpful in improving the reliability of patient assessment, and thereby assist in the assignment of appropriate treatment. Fine needle aspiration (FNA) biopsy is a proven effective procedure for the diagnosis of malignant diseases of the human breast (Orell *et al.*, 1986), and a simultaneous evaluation of FN aspirates for the heterogeneity of oestrogen receptor expression within the cancer cell population would yield an early assessment of prognosis without recourse to surgery. The newly emerging technique of video image analysis promises to remove much of the subjectivity integral to semi-quantitative assessments of immunocytochemical staining (Charpin *et al.*, 1986).

Our aims in performing this study were: (1) to develop an image analysis method to measure objectively heterogeneity of ER expression by breast cancer nuclei in fine needle aspirate; and (2) to compare the results from the image analysis of FNA with the biochemically determined ER concentration of the corresponding surgical biopsy for all patients in the study.

### Materials and methods

### *Tissues*

Breast carcinoma tissue was collected for this study from 35 sequential surgical patients at Flinders Medical Centre. All patients had primary breast cancer disease, and had received no preoperative treatment. Biopsies were transported on ice to the laboratory within 10 minutes of removal in theatre. A representative sample of approximate dimension 1 × 10 × 0.5 cm was trimmed of fat for the hormone receptor analyses.

Correspondence: D.J. Horsfall.
Received 20 July 1988; and in revised form, 26 September 1988.

### *Preparation and ER staining of fine needle aspirates*

The tumours, originally diagnosed as malignant by FNA in the breast clinic, were reaspirated on an ice-cold aluminium block to provide source material for the video image analysis. A 26 gauge needle with negative pressure applied was passed through the surgical biopsy a minimum of six times at varying angles and sites to ensure a representative sample was taken, and the material collected was dispersed into a 0.5 ml aliquot of RPMI 1640 medium (Gibco, NY) by vortexing. Aspirated cells were then deposited on to poly-L-lysine (Sigma, St Louis, MO) coated slides by cytocentrifuge. The tumour cytospins were fixed and stained for the presence of ER using an ERICA kit (Abbott Diagnostic Labs, Chicago, IL) according to the manufacturer's instructions. The cytospins were frequently stored between the fixation and the staining steps for 24–48 h at −25°C in the glycerol sucrose buffer recommended by the ERICA instruction sheet. Counterstaining of the nuclei was achieved using weak Haematoxylin (1:1 diluted Lillie Mayer's Haematoxylin) for 30 s. The specimens were then dehydrated and mounted for light microscopy. It is important to stress that, before staining, care should be taken to prevent drying of the specimen, since this results in a decrease in receptor antigenicity.

### *Biochemical quantitation of oestrogen receptor*

The surgical biopsy was snap frozen immediately after needle aspiration and stored at −70°C for 24–48 h. Quantitation of ER was by radioligand binding in a multipoint dextran-coated charcoal assay with Scatchard analysis, as reported previously (Horsfall *et al.*, 1986). Receptor concentrations were expressed as fmol mg$^{-1}$ cytosol protein. Tumour cytosols with an oestrogen receptor concentration equal to or greater than 10 fmol mg$^{-1}$ protein were graded as ER positive in this study.

### *Video image analysis of ERICA stained aspirates*

The video image analytical system used in this study was developed in our laboratories (Jarvis, 1986, 1987, 1988). In brief, the system comprises a microscope, solid state camera, video digitiser, microcomputer and digitiser tablet (Jarvis, 1988). The image analysis software permits selection and scoring of individual tumour cells through editing of debris, overlapping or touching nuclei, stromal cells or any other undesired feature. The editing functions are performed by a moving cursor operated manually through the linked digitiser pad. Criteria for assigning malignant versus benign to cellular elements were based on standard cytological features, including nuclear/cytoplasmic ratio, nuclear pleomorphism and nuclear irregularity.

Sequential fields were systematically examined using a ×40 objective and a ×5 camera eyepiece to assess a minimum of 250 nuclei from both the primary ER monoclonal antibody-stained and the control antibody-stained cytospins. Each field was analysed alternately at two wavelengths using interference filters of 550 nm (green) 50 nm bandwidth, and 436 nm (blue) 16 nm bandwidth. The 550 nm image allowed detection and editing of the total number of nuclei present by virtue of the haematoxylin counterstain. Those stained with the red–brown diaminobenzidine (DAB) deposit were isolated by the 436 nm filter, which reduced the absorbance contribution of the haematoxylin dye to an undetectable level. Integrated optical density (IOD) measurements at 436 nm were therefore related to the total amount of DAB deposit in each nucleus. To enable comparisons to be made between expression of oestrogen receptor in terms of either total content per nucleus or mean concentration per nucleus, the integrated optical density measurement for each nucleus was divided by its area to give an average optical density (OD) measurement for that nucleus.

Various strategies were employed for calculating a positivity index for nuclear ER from the integrated density and average density measurements of nuclear DAB. Because control antibody-stained nuclei often contain measurable levels of DAB, which result from endogenous peroxidase activity and non-specific antibody binding, each strategy was designed to compensate for this background staining level.

1. A positivity index based on nuclear ER content was calculated as the percentage of nuclei in the monoclonal antibody-stained cytospin above a threshold point denoting the background staining level. To determine the threshold point for the background, the absolute upper limit of the range of IOD values in the control preparation gave a poor estimate due to variable presence of low frequencies of densely stained nuclei. Consequently, the threshold was calculated as the upper 95th percentile point of the frequency distribution of IOD measurements of nuclei of the control. This provided an IOD value for which 95% of the nuclei below this point in the positive preparation would have a background, or negative, staining level. The positivity index was then calculated as the percentage of nuclei in the positive preparation above this threshold value (Figure 1). The 5% error involved in scoring some of the negative nuclei as positive is negated by a similar overlap of positive nuclei scored as negative. The degree of this overlap and relative proportions of incorrectly scored nuclei could not be determined.

2. A similar positivity index expressed as a percentage of positive nuclei above an IOD threshold was calculated as for the percentile method. However, the threshold IOD value was calculated as a 95% probability level for the distribution of frequencies of nuclear IOD in the control preparation. Below this point, a nucleus in the positive preparation could be identified as negative with 95% confidence. The selection criteria were therefore based on statistical assumptions rather than arbitrary choice of cut-off point as for the percentile method. In each control preparation, the assumption was made that the distribution of nuclear IOD was normal, although most histograms indicated skew (e.g. Figure 1). A positivity index of the percentage of nuclei in the positive preparation was calculated as for the percentile method.

3. A positivity index based on integrated density of staining (ER content) was calculated as the mean IOD of DAB per nucleus. The level of background staining was compensated for by subtracting the mean IOD of the nuclei in the control preparation from the mean IOD of the nuclei in the positive preparation. The positivity index, expressed in the pixel units of the image analyser, was termed the $IS_{SCORE}$.

4. A similar positivity index was calculated from the mean IOD of DAB per nucleus. However, the background staining was compensated for by dividing the mean IOD of nuclei in the positive preparation by the mean IOD of nuclei in the control. The positivity index, expressed as the ratio of positive DAB IOD and negative DAB IOD was termed the $ID_{SCORE}$.

5. A positivity index based on staining density (ER concentration) was calculated from the mean optical density of DAB per nucleus. The background staining was compensated for by subtracting the mean optical density of the nuclei in the control preparation from that of the positive preparation. The positivity index was termed the $S_{SCORE}$.

6. A similar positivity index was calculated from the mean optical density of DAB staining per nucleus. However, the background staining was compensated for by dividing the mean optical density of stain in nuclei of the positive preparation by the mean optical density of stain in nuclei of the control. The positivity index was termed the $D_{SCORE}$.

*Statistical analyses*

Association between the ER concentrations of the patient cancer series as measured by the radioligand binding assay and the various video image analysis indices was tested by Pearson's correlation coefficient. The lines of best fit for the comparisons of ER determined by various methods were calculated by least squares analysis. Analysis of the concordance rate in determination of receptor status was performed using $\chi^2$ tables.

**Table I** Oestrogen receptor values for individual primary breast cancer patients as determined by DCC and image analysis

| *Patient no.* | *DCC*[a] | *Percentage ER-positive*[b] | *Percentage ER-positive*[c] |
|---|---|---|---|
| 1 | 219 | 63 | 61 |
| 2 | 37 | 8 | 7 |
| 3 | 156 | 26 | 39 |
| 4 | 62 | 51 | 53 |
| 5 | 13 | 13 | 11 |
| 6 | 347 | 87 | 85 |
| 7 | 225 | 8 | 6 |
| 8 | 238 | 5 | 5 |
| 9 | 214 | 32 | 33 |
| 10 | 336 | 38 | 30 |
| 11 | 73 | 3 | 6 |
| 12 | 35 | 1 | 2 |
| 13 | 207 | 9 | 14 |
| 14 | 67 | 34 | 29 |
| 15 | 180 | 29 | 37 |
| 16 | 158 | 57 | 55 |
| 17 | 263 | 51 | 54 |
| 18 | 65 | 33 | 34 |
| 19 | 88 | 37 | 33 |
| 20 | 34 | 34 | 35 |
| 21 | 31 | 3 | 3 |
| 22 | 45 | 27 | 24 |
| 23 | 64 | 16 | 15 |
| 24 | 372 | 57 | 49 |
| 25 | 100 | 24 | 23 |
| 26 | 42 | 5 | 8 |
| 27 | 218 | 22 | 24 |
| 28 | 0 | 0 | 0 |
| 29 | 0 | 2 | 2 |
| 30 | 0 | 0 | 0 |
| 31 | 0 | 0 | 0 |
| 32 | 0 | 0 | 0 |
| 33 | 0 | 0 | 0 |
| 34 | 0 | 0 | 0 |
| 35 | 0 | 0 | 0 |

All patients were infiltrating ductal carcinoma not otherwise specified (NOS) with the exception of patients 11 (invasive lobular) and 29 (medullary).

[a]ER fmol $mg^{-1}$ cytosol protein by dextran coated charcoal assay. [b]Using 95th percentile of control for background binding. [c]Using 95% probability level for background binding.

A minimum of 250 nuclei were assessed by image analysis on both primary antibody and control antibody-stained cytospins.

**Figure 1** Examples of image analysis profiles of ER immunocytochemical staining for four patients. Filled bars indicate frequency of nuclei staining with defined integrated optical density of diaminobenzidine (DAB) on primary monoclonal antibody-stained cytospins. Open bars indicate frequency of stained nuclei on control antibody-stained cytospins. The arrow in each case indicates the threshold of positive immunostaining calculated by the 95th percentile method for the control slide. Percentage ER positivity: a, 63%; b, 87%; c, 51%; d, 0%.

### Results

The primary breast cancer patients in this study were predominantly of NOS infiltrating ductal carcinoma histopathology (Table I). A breakdown of the oestrogen receptor quantitation by the DCC assay and the percentage ERICA-positive nuclei using the 95th percentile and 95% probability level methods to account for background binding is also presented for the individual patients in Table I. Figure 1 comprises several representative histograms of ERICA-stained needle aspirates employing the 95th percentile for background staining and illustrating the variability of nuclear staining between individual cancers. The histogram for patient no. 1 is illustrated in Figure 1a and indicates two major populations of nuclei on the primary antibody-stained slide, one corresponding to the control population of unstained nuclei and an additional population of ER-containing nuclei. Approximately two-thirds of the nuclei stained positive for ER in this patient, with high levels of ER also detected by the biochemical assay (219 fmol mg$^{-1}$ cytosol protein). In patient no. 6 (Figure 1b) almost 90% of the nuclei contained ER, with approximately two-thirds staining for low levels and one-third for high levels of ER. This patient was also among the higher biochemically determined values of ER for this series of patients (347 fmol mg$^{-1}$). Patient no. 4 (Figure 1c) was an interesting case. No discrete peak of ER-staining nuclei was apparent but instead a population of tumour nuclei with broad heterogeneity in expression of ER. Fifty per cent of the tumour nuclei stained positively for ER. Figure 1d illustrates the histogram for patient no. 32, where no nuclei of the primary antibody-stained aspirate had DAB levels greater than the nuclei of the control antibody-stained aspirate. The biochemical determination for this biopsy was also negative (0 fmol mg$^{-1}$).

The relationship between the number of ER-positive nuclei for each individual case, as calculated using 95th percentiles

**Figure 2** Association between percentage ER-positive nuclei for all cases, calculated by 95th percentiles and 95% probability levels ($r = 0.98$; $P < 0.0005$). 95% confidence limits for the line of best fit are indicated.

and 95% probability levels, is shown in Figure 2. The line of best fit was calculated and indicated a high correlation ($r = 0.98$; $P < 0.0005$).

The frequency distribution of tumours with varying percentages of ER-positive nuclei using 95th percentiles is illustrated in Figure 3. The frequencies are not normally distributed about a mean but appear to be evenly distributed, predominantly below 40% ER-positive cells. Only six of the 28 ER-positive cancers had greater than 40% ER-positive nuclei.

**Figure 3** Frequency distribution of percentage ER-positive nuclei for all patient cancers.

**Table II** Association between ER determination by DCC and image analysis indices

| Image analysis index | Pearson's correlation coefficient, r | Probability level, P |
|---|---|---|
| IOD 95th percentile | 0.617 | <0.001 |
| IODC 95% probability level | 0.590 | <0.001 |
| $IS_{SCORE}$ | 0.383 | <0.05 |
| $ID_{SCORE}$ | 0.062 | =0.74 |
| $S_{SCORE}$ | 0.345 | =0.07 |
| $D_{SCORE}$ | 0.017 | =0.57 |

**Figure 4** Association between percentage ER-positive nuclei by image analysis using 95th percentiles, and ER concentration by radioligand binding assay (DCC) for all patient cancers ($r = 0.77$; $P < 0.0005$). 95% confidence limits for the line of best fit are indicated.

The association between ER determination by the standard DCC method and the various image analysis indices is indicated in Table II. Significant associations ($P < 0.001$) were obtained by the 95th percentile and 95% probability level methods for determining percentage of tumour nuclei with an IOD for ER above that of background. Similarly, subtraction of mean IOD for the control population of nuclei from the IOD of the ERICA primary antibody-stained nuclei ($IS_{SCORE}$) yielded a statistically significant association. However, image analysis indices based either on a division of mean IOD for the primary antibody-stained population by the control population IOD, or on staining intensity (mean optical density rather than integral optical density) of the nucleus, failed to show significant association with the ER value determined by the DCC method (Pearson's correlation coefficient).

The relationship between the percentage of ERICA-positive nuclei in the needle aspirate, as determined by video image analysis using the 95th percentile method to account for control staining, and the biochemical quantification of the surgical biopsy for all 35 patients was examined in Figure 4. The line of best fit by the least squares method was calculated and indicated a high correlation ($r = 0.77$; $P < 0.0005$). The assignment of threshold values of 10 fmol mg$^{-1}$ for the DCC receptor analysis and 5% ER-positive cells by image analysis enabled determination of the sensitivity (true positive ratio), specificity (true negative ratio), concordance and predictive values for positivity and negativity (McCarty *et al.*, 1985). Using the thresholds outlined above, the results shown in Tables III and IV were obtained. Concordance of receptor status was 91%. Discordance was seen in only three cases where tumours were ER-positive by the biochemical assay and negative by ER video image analysis, yielding a predictive value for positive of 100% and predictive value for negative of 73%. Sensitivity equalled 89% and specificity equalled 100%. This association between ER positivity of breast cancers as analysed by DCC and ERICA (Table III) was significant ($\chi^2 = 23.17$, $P < 0.001$).

**Table III** Categorisation of receptor status of breast cancer cases as analysed by DCC and image analysis

| | | Saturation analysis of cytosolic radioligand binding (DCC) | |
|---|---|---|---|
| | | Positive[a] | Negative |
| Image analysis of fine needle aspirate | Positive[b] | 24 | 0 |
| | Negative | 3 | 8 |
| | $\chi^2 = 23.17$ $P < 0.001$ | | |

Threshold values: [a]$\geq$10 fmol mg$^{-1}$. [b]$\geq$5% ER-positive nuclei.

**Table IV** Sensitivity, specificity and predictive value of image analysis compared to biochemical assay (DCC)

| Concordance[a] | Sensitivity[b] | Specificity[c] | Predictive value + [d] | Predictive value − [e] |
|---|---|---|---|---|
| 32/35 (91%) | 24/27 (89%) | 8/8 (100%) | 24/24 (100%) | 8/11 (73%) |

Threshold value for ER positivity: Biochemical assay $\geq$ 10 fmol mg$^{-1}$; Image analysis $\geq$5% ER-positive nuclei.
[a]Concordance = (TP + TN)/(TP + FP + TN + FN) × 100%.
[b]Sensitivity = TP/(TP + FN) × 100%.
[c]Specificity = TN/(TN + FP) × 100%.
[d]Predictive value of a positive result = TP/(TP + FP) × 100%.
[e]Predictive value of a negative result = TN/(TN + FN) × 100%.
TP, true positive; FP, false positive; TN, true negative; FN, false negative.

### Discussion

In this study of breast cancer patients the ERICA assay was shown to have a concordance rate of 91% with the DCC assay. Similar concordance rates have been reported by Charpin *et al.* (1986), King *et al.* (1985), McCarty *et al.* (1985) and Pertschuk *et al.* (1985) (88, 84, 91 and 86% respectively). Compared with the DCC assay, image analysis had a predictive value for a positive result of 100%, but only 73% for predicting negative results. This was attributed to the 'incorrect' negative classification of several DCC-positive tumours when an arbitrary ERICA threshold of 5% positive nuclei was used. Reduction of this arbitrary threshold to 3% positive nuclei improved the prediction of negative results to

91%. To date there have been no large studies of the relationship between ERICA results and response to endocrine therapies in patients with advanced disease. McClelland *et al.* (1986) demonstrated that ERICA predicted outcome to therapy in 81% of 47 courses of treatment (compared with 72% for the DCC assay). Similar results showing the improved predictive ability of ERICA over the DCC assay were also demonstrated by Pertschuk *et al.* (1985) (80% versus 58%).

The majority of research groups performing ER immunocytochemistry on frozen sections or fine needle aspirate have employed a histological scoring index based on a combination of visual analyses of the intensity of immunostaining and proportion of immunostained nuclei (King *et al.*, 1985; Pertschuk *et al.*, 1985; Azavedo *et al.*, 1986; Flowers *et al.*, 1986; Jonat *et al.*, 1986). This approach can lead to a high degree of subjectivity in the assessment of staining intensity and hence the proportion of stained nuclei, and this is difficult to control. The intra- and inter-observer variation in assessments can be minimised by the use of a computerised system of image analysis. To date there have been few published reports of video image analysis applied to the estimation of ER heterogeneity in breast cancer nuclei (Charpin *et al.*, 1986; Sklarew & Pertschuk, 1987; Franklin *et al.*, 1987). With these systems, background staining in control populations of cells due to endogenous peroxidase and non-specific antibody binding can be readily quantitated by the computer system, thereby improving the reliability of the measurements. In this study we considered several ways to discriminate and compensate for background levels of staining. The resultant positivity indices for each tumour were then compared with the ER concentrations derived using the DCC assay – currently the 'gold standard' for ER quantitation. It appeared that the most reliable indices for measuring ER-related deposition of stain in the needle aspirates were those employing 95th percentiles or 95% probability levels to identify for each specimen a unique nuclear integrated optical density, below which staining absorbance could be determined to be of control level. Nuclei with integrated optical densities above this level were deemed ER-positive and were graded accordingly. Indices based solely on intensity of staining in fine needle aspirate were unrelated to the biochemically determined ER concentration. While the index based on a 95% probability value for the control staining level is an acceptable statistical technique, the alternative index based on percentiles proved to be equally effective (Figure 2), possibly due to the high skew of the nuclear IOD distributions. The percentile index requires minimal computation and for this reason is the technique of choice.

Quantitation of ER in fine needle aspirate of breast cancer by video image analysis requires different assumptions to be made than when analysing frozen sections. If one wishes to measure the IOD of individual nuclei in frozen sections, extensive programming is required to account for the fact that the majority of cancer nuclei are not sectioned through the greatest diameter of the cell – even if they all could be assumed to be of the same size and shape. As observed by Sklarew & Pertschuk (1987), an important consideration in densitometry of sectioned material is the effect of nuclear volume distribution on the observed receptor concentration (mean optical density). Using nuclear volumes derived from projected nuclear areas, these workers determined that the presence of well-separated concentration peaks could not be ascribed to differences in ER content, but were dependent on variations in nuclear volume. Furthermore, in sectioned material estimates of mean optical density were decreed as 'not always meaningful', which would agree with our observation for fine needle aspirate that mean optical density measurements and oestrogen receptor quantitation by DCC are not associated. While mathematic considerations of total nuclear volume as determined for sectioned nuclei do not apply to needle aspirates since the whole nucleus is available for measurement, intensity of staining measurements will be highly subject to nuclear area variations, due to technical artefacts of cytocentrifugation. The statistical association of integrated optical density measurements with the biochemical ER determination for FN aspirates indicates, however, that total ER content is far more important than ER concentration. Hence, for assessment of FN aspirates at least, greater accuracy in reporting will be achieved using indices based on percentage ER-positive nuclei determined by image analysis of nuclear IOD.

In one only wishes to determine whether a tumour is ER-positive, then it appears that there is a strong correlation between the ERICA and DCC techniques as indicated by the concordance rates (Table IV), but when individual comparisons are made on single cancers significant quantitative discrepancies can be observed, which need to be explained (Figure 4). For example, why should two cancers with 50–60% ER-positive cells have individual DCC values of 62 $\text{fmol mg}^{-1}$ and 372 $\text{fmol mg}^{-1}$; or conversely two tumour cytosols with DCC values of approximately 200 $\text{fmol mg}^{-1}$ have values as disparate as 9% and 53% ER-positive cells? Part of the answer may lie in cytosol preparation, which leads to an averaging of ER concentration within the extracted tissue. DCC quantitation is heavily dependent on tumour cellularity and malignant to benign cell ratio. Theoretically then, one might expect significant variability to be generated by the DCC assay and most investigators are aware of this problem. Presently there is less certainty regarding variability arising from immunocytochemical detection of ER. Currently, studies are in progress within our laboratory to identify and eliminate potential sources of variability in the preparation of immunocytochemically stained cytospins of fine needle aspirate. Such studies will enhance the acceptibility of ERICA and image analysis of ER heterogeneity, and allow this new technology to supercede the more complex and less economic DCC assay.

Technically, at the present time all image analysis indices are reliant on relative quantitation of DAB deposition and no system exists for absolute quantitation of the number of molecules of ER within individual cancer nuclei. However, it is possible that systems based on microspheres with chemically coupled receptor, or cloned cell lines with constitutive synthesis of ER from amplified gene constructs, may be developed to assist in this quantitation.

One further point, which relates to the biological nature of breast cancer disease, is raised by the frequency distribution of percentage ER-positive nuclei in Figure 3. In this study the majority (79%) of breast cancers examined had less than 40% ER-positive nuclei. This means that even in ER-positive cancers, the majority of cells may not express oestrogen receptors. Whether this absence of receptors is due to the growth phase of the ER-positive cells, selection artefacts by FNA, or a true ER-negative subpopulation needs to be closely examined as this heterogeneity would have direct prognosis implications for long-term clinical response to endocrine therapy.

In conclusion, this study has demonstrated that video image analysis of ERICA-stained nuclei obtained by fine needle aspiration is an appropriate method for assigning ER status in breast cancer. Furthermore this method is easy and economic to perform, requiring only some cytological experience in the discrimination of tumour and benign cellular elements.

The authors would like to express their appreciation to Mr Paul Stoll for his indefatigable computer programming and to Ms Kimberly Goldsmith for her artwork. Our thanks also go to Professor V.R. Marshall and Dr R. Seshadri for reviewing the manuscript and to Mrs Y. Rigos for assisting in its preparation. This study was supported by grants from the National Health and Medical Research Council of Australia, the Anti-Cancer Foundation of the Universities of South Australia and the Flinders Medical Centre Research Foundation. This study was presented in part at the Australian Society for Medical Research, Sydney, December 1986, and at the International Breast Cancer Research Association, Miami, March 1987.

### References

AZAVEDO, E., BARAL, E. & SKOOG, L. (1986). Immunohistochemical analysis of estrogen receptors in cells obtained by fine needle aspiration from human mammary carcinomas. *Anticancer Res.*, **6**, 263.

CANT, E.L.M., HORSFALL, D.J. & KEIGHTLEY, D.D. (1985). Value of hormone receptors in the management of breast cancer. 1. Advanced breast cancer. *Aust. NZ J. Surg.*, **55**, 121.

CHARPIN, C., MARTIN, P.-M., JACQUEMIER, J., LAVANT, M.N., POURREAU-SCHNEIDER, N. & TOGA, M. (1986). Estrogen receptor immunocytochemical assay (ER-ICA): Computerised image analysis system, immunoelectron microscopy, and comparisons with estradiol binding assays in 115 breast carcinomas. *Cancer Res.*, **46**, 4271s.

FLOWERS, J.L., COX, E.B., GEISINGER, K.R. & 4 others (1986). Use of monoclonal antiestrogen receptor antibody to evaluate estrogen receptor content in fine needle aspiration breast biopsies. *Ann. Surg.*, **203**, 250.

FRANKLIN, W.A., BIBBO, M., DORIA, M.I. & 4 others (1987). Quantitation of estrogen receptor content and Ki-67 staining in breast carcinoma by the MicroTICAS image analysis system. *Anal. Quant. Cytol. Histol.*, **9**, 279.

HORSFALL, D.J., TILLEY, W.D., ORELL, S.R., MARSHALL, V.R. & CANT, E.L.M. (1986). Relationship between ploidy and steroid hormone receptors in primary invasive breast cancer. *Br. J. Cancer*, **53**, 23.

JARVIS, L.R. (1986). A microcomputer system for video image analysis and diagnostic microdensitometry. *Anal. Quant. Cytol. Histol.*, **8**, 201.

JARVIS, L.R. (1987). Adapting video technology for quantitative microscopy. *Acta Stereol.*, **6**, 299.

JARVIS, L.R. (1988). Microcomputer video image analysis. *J. Microsci.*, **150** (in the press).

JONAT, W., MAASS, H. & STEGNER, H.E. (1986). Immunohistochemical measurement of estrogen receptors in breast cancer tissue samples. *Cancer Res.*, **46**, 4296s.

KING, W.J., DESOMBRE, E.R., JENSON, E.V. & GREENE, G.L. (1985). Comparison of immunocytochemical and steroid-binding assays for estrogen receptor in human breast tumors. *Cancer Res.*, **45**, 293.

McCARTY, K. JR, SNOWHITE, E., COX, E., MILLER, L. & McCARTY, K. SR (1985). Monoclonal antibodies against estrogen receptor: Specificity, sensitivity and potential applications. In *Monoclonal Antibodies and Breast Cancer*, Ceriani, R.L. (ed) p. 190. Martinus Nijhoff: Boston.

McCLELLAND, R.A., BERGER, U., MILLER, L.S., POWLES, T.J. & COOMBES, R.C. (1986). Immunocytochemical assay for estrogen receptor in patients with breast cancer: Relationship to a biochemical assay and to outcome of therapy. *J. Clin. Oncol.*, **4**, 1171.

McGUIRE, W.L. (1987). Prognostic factors for recurrence and survival in human breast cancer. *Breast Cancer Res. Treat.*, **10**, 5.

ORELL, S.R., STERRETT, G.R., WALTERS, M.N. & WHITAKER, D. (1986). *Manual and Atlas of Fine Needle Aspiration Cytology*. Churchill Livingstone: Edinburgh.

PERTSCHUK, L.R., EISENBERG, K.B., CARTER, A.C. & FELDMAN, J.G. (1985). Immunohistologic localisation of estrogen receptors in breast cancer with monoclonal antibodies. Correlation with biochemistry and clinical endocrine response. *Cancer*, **55**, 1513.

SKLAREW, R.J. & PERTSCHUK, L.P. (1987). Quantitation of the immunocytochemical assay for estrogen receptor protein (ER-ICA) in human breast cancer by television imaging. *J. Histochem. Cytochem.*, **35**, 1253.

THORPE, S.M., ROSE, C., RASMUSSEN, B.B. & 6 others (1986). Steroid hormone receptors as prognostic indicators in primary breast cancer. *Breast Cancer Res. Treat.*, **7**, suppl., 91.

WILLIAMS, M.R., TODD, J.H., ELLIS, I.O. & 6 others (1987). Oestrogen receptors in primary and advanced breast cancer: An eight year review of 704 cases. *Br. J. Cancer*, **55**, 67.